# Infant Social Withdrawal Behavior: A Key for Adaptation in the Face of Relational Adversity

**DOI:** 10.3389/fpsyg.2022.809309

**Published:** 2022-06-20

**Authors:** Sylvie Viaux-Savelon, Antoine Guedeney, Alexandra Deprez

**Affiliations:** ^1^Institut des Sciences Cognitives Marc Jeannerod, UMR 5229 CNRS, University Hospital Croix Rousse, HCL, Lyon, France; ^2^Groupe Hospitalier Universitaire AP-HP Nord, Université de Paris, Paris, France; ^3^Institut de Psychologie Laboratoire de Psychopathologie et Processus de Santé, LPPS, EA 4057, Université de Paris, Paris, France; ^4^B-Families Sarl, Luxembourg, Luxembourg

**Keywords:** infant sustained social withdrawal behavior, infant depression, parent-infant dys-synchrony, development of inter subjectivity, defensive process in the face of relational adversity, attachment strategies, polyvagal theory, translational research in social neurosciences

## Abstract

As a result of evolution, human babies are born with outstanding abilities for human communication and cooperation. The other side of the coin is their great sensitivity to any clear and durable violation in their relationship with caregivers. Infant sustained social withdrawal behavior (ISSWB) was first described in infants who had been separated from their caregivers, as in Spitz's description of “hospitalism” and “anaclitic depression.” Later, ISSWB was pointed to as a major clinical psychological feature in failure-to-thrive infants. Fraiberg also described freezing behavior as one of the earliest modes of infant defense in the face of adverse situations threatening the infant's ability to synchronize with caregivers. We hypothesize that ISSWB behaviors are associated with poor vagal brake functioning and that an impaired social engagement system is induced by an impoverished and/or dangerous environment. Recent research using animal models highlight the neurobiology and the genetics of the social Approach/Withdrawal Behavior in infants. The present paper is therefore a plea for social withdrawal behavior to be attributed a more important role as a major psychological defensive mechanism in infancy, and for research into early development and early intervention to make more practical and theoretical use of this concept, thus decreasing the challenge of translation in social neurosciences. This work presents several situations involving developmental hazards in which assessment of ISSWB by means of the Alarm Distress Baby Scale (ADBB) has proven useful, i.e., malnutrition, effects of major maternal depression and or traumatization, assessing social withdrawal in infants with an chronic organic illness (congenital heart disease, Prader-Willi syndrome, cleft lip and/or palate Prader-Willy syndrome, Fetal alcohol syndrome) or assessing ISSWB in out of home placed infants during parental visitation. Relationships between ISSWB and other biophysiological behavioral systems are discussed, particularly links with attachment processes and Porges's polyvagal theory.

## Introduction

What are the neural mechanisms that improve or inhibit social interaction? Recently, researchers have begun to address these questions with human neuroimaging studies seeking to map various aspects of higher order processing of social information and even develop computational models of the neural basis of social cognition (Bartz and Hollander, 2006). If human neuroimaging studies can describe the cortical patterns, mechanistic studies are still mostly the domain of animal research. So far, clinical description, research in infancy development, early psychopathology and animal research have had little in common (Insel, 2010), except for some intermittent cases. However, identification of common mechanisms between early modes of defense and developmental processes might be a fruitful way of research since animal models are the main key for exploring early brain development in terms of physiology and genetics.

Because they are born with no ability to protect themselves or move independently, human babies take exceptionally long to both mature and develop psychologically. Humans are cooperative breeders (Burkart et al., 2009), which means that every adult can take care of the young of their species (Hrdy, 1999). Caregiving and attachment systems are intertwined and have therefore followed parallel developmental pathways throughout evolution (Bowlby, 1973). Human babies are born with some very effective social abilities, with great interest in human communication, and are endowed with some “core knowledge” (Spelke, 2000). However, this comes at a price, given the great sensitivity of the human infant toward any clear and durable violation in the relationships with their caregivers. Several behavioral systems have developed within the evolutionary process to help human infants develop their cooperative and social abilities and help them survive the hazards of the relationship with their caregivers. Social withdrawal behavior is a normal mechanism used by the infant to regulate the flow of social interaction (Brazelton et al., 1974; Cohn and Tronick, 1983). It appears to be among the first defense mechanisms infants may use in the face of relational adversity when protestation fails (Fraiberg, 1982; Guedeney et al., 2013). Therefore, it seems that infant sustained social relational withdrawal (ISSWB) is among the first defense mechanisms that infants can use. This makes ISSWB a valuable early alarm signal and a behavior worth studying transversely across developmental disciplines to understand its relationships with other behavioral systems. The animal models of social engagement and social withdrawal may help us map its neurological organization as well as its genetic pattern.

We will firstly address ISSWB and its relationships with several conditions in early infancy, where babies must cope with environmental or somatic challenges, such as autism spectrum disorder (ASD), Prader-Willi syndrome (PWS), fetal alcoholism syndrome (FAS), major depressive disorders or maternal post traumatic experiences, home placed infants during parental visitation, palate and cleft syndrome, effect of early cardiac surgery on infants. In a second part, we will discuss the Relationships between ISSWB and other biophysiological behavioral systems particularly links with attachment processes and the role for ISSWB as a “vagal brake” in terms of Porges's theory.

## Why Is Social Withdrawal Behavior in Infants Important to Consider, Both Theoretically and Clinically?

### A Brief History of the Recognition of Infant Sustained Social Withdrawal Behavior

The term social withdrawal is used increasingly more in the clinical study of infancy, but without a clear definition. Rene Spitz (Spitz, 1946) was among the first to use this term in his famous clinical description of anaclitic depression. Engel and Schmale (1972), described sustained social withdrawal behavior as a defense mechanism in an 14-month-old marasmic, developmentally retarded infant, Monica, leading to the description of social withdrawal behavior as an energy conservation mechanism in infants faced with affective deprivation (Engel and Schmale, 1972). Observations of young children separated from their caregivers led Bowlby (1973) to describe a three-stage emotional reaction among young children: protest, despair, and withdrawal behavior—and eventually detachment in cases of prolonged separation, which led him to propose a radically new way of describing early infant and child psychopathology (Ainsworth and Bowlby, 1991). Based on extensive clinical experience, Fraiberg (1982) described a group of pathological defenses observed in infants between 3 and 18 months of age who had experienced severe danger, threats or deprivation. These early defenses, “avoidance,” “freezing,” and “fighting,” according to Fraiberg, obviously derived from a biological repertoire classified by Bowlby into the “fear behavioral system” (Bowlby, 1973). Following the same line of thought, some North American pediatricians have described the psychological state of some infants in conditions of malnutrition. These failure to thrive (FTT) infants display a specific alteration of the approach/withdrawal behavior. This enabled the building of the first approach/withdrawal scale in infants by Powell and Low (1983). In tropical countries, pediatricians had long turned their attention to the intense and prolonged social withdrawal behavior in infants with severe forms of protein-energy malnutrition, such as kwashiorkor (Williams, 1938; Geber and Dean, 1956), leading to the recognition of the links between this disorder and maternal postpartum depression and with insecure and disorganized attachment (Guedeney, 1995; Mc Mahan True et al., 2001; Van den Heuvel et al., 2017). Clearly, FTT and the different kinds of malnutrition are associated with specific developmental delay, sharing a common high level of ISSWB.

### Infant Sustained Social Withdrawal Behavior as a First Line of Defense in the Face of Major and Repeated Failures of Synchronization Within Infant-Caregiver Relationships

Because of human infants' extreme immaturity at birth, infants depend on the caregiving context for relatively long periods of time, during which they require specific environmental inputs for the regulation of their biological and behavioral systems (Bowlby, 1973). The most important source for the provision of such inputs is the mother's body and physical presence, or those of any available caregiver. Maternal proximity and interactive behaviors provide an “external regulatory” function for the organization of neurobiological, sensory, perceptual, emotional, physical, and relational systems (Hrdy, 1999). Development is thus firmly grounded in relationships, and this initial dependence of the infant on the mother's body opens up a lifelong neurobiological possibility that one person can provide an “external regulatory” function in the physiological systems of another through timely adaptation to distress and social cues. Indeed, studies on maternal deprivation have long shown that maternal absence in humans and mammals is associated not only with physiological dysregulation but with social withdrawal, apathy, and disengagement (Spitz, 1946; Zeanah et al., 2000).

A key element in early development is arguably the parent-infant triad's ability to synchronize with one another, particularly during the first 18 months of the infant's life. Synchrony is, according to Feldman (2007), the “co-regulatory” experience in attachment relationships that provides the foundation for the child's later capacity for intimacy, symbol use, empathy, and the ability to read the intentions of others (Feldman, 2007, p. 330). The synchrony of the relationship between mother and child as well as between father and child is therefore a major determinant of all infant developmental outcomes (Feldman, 2007; Leclere et al., 2014). Feldman (2007) discussed synchrony for the caregiver-infant dyad as a temporal and organizing feature of the relationship. Increased or sustained social withdrawal reaction in infants can be observed in suboptimal parent-infant interactions, such as those observed between severely depressed mothers or mothers with borderline personality disorder and their infants, where mismatches and misreading of the infant's cues occur very frequently, preventing the appropriate response. An infant's “depressed” style of interacting can be carried over to other relationships as well and can be apparent even when the infant interacts with a non-depressed adult (Field et al., 1988; Weinberg and Tronick, 1996; Tronick, 2007).

Maternal and child factors both contribute to synchrony between parent and child, and child withdrawal and maternal depression are associated with failed synchrony and the associated developmental psychopathology. Feldman (2007) stressed the importance of infant sustained withdrawal behavior as a sign of dysregulation in parent-infant synchrony. The infant's reactions to interruption or to the violation of his expectations in their interactions are both obvious and durable in the “Still-Face” paradigm (Cohn and Tronick, 1983; Weinberg and Tronick, 1996). It is also why ISSWB is worth being detected at an early stage, considering its potential developmental effects on cognition, social relationships and the development of intersubjectivity as described by Trevarthen and Aitken (1999).

### Using the Alarm Distress Baby Scale to Screen for Infant Sustained Social Withdrawal Behavior

The ADBB detects social withdrawal in infants aged 2–24 months. It is an observer-rated screening tool assessing social behavior across eight items: (1) facial expressions, (2) eye contact, (3) general level of activity, (4) self-stimulating gestures, (5) vocalizations, (6) briskness of response to stimulation, (7) relationship, and (8) attraction (Guedeney and Fermanian, 2001). Infant social withdrawal is characterized by less frequent eye contact, fewer emotional displays, less vocalizations, a decreased level of activity, and possibly by increased self-stimulation and delayed reaction time (Guedeney et al., 2013). Let us not forget that social withdrawal within a certain range is a normal part of caregiver-infant interactions allowing the infant to self-regulate (Brazelton et al., 1974; Field et al., 1988). In contrast, sustained social withdrawal may have adverse effects on child development, since this behavior limits the infant's access to the social learning environment (Guedeney et al., 2013). Indeed, studies have shown that social withdrawal in infancy is associated with less optimal outcomes within several developmental domains, such as emotional and behavioral disorders (Guedeney et al., 2014; Zhou et al., 2021) and poorer cognitive and language development (Milne et al., 2009; Guedeney et al., 2017; Smith-Nielsen et al., 2019).

Several validations of the ADBB in different countries and cultures yielded the same cut-off scores (Guedeney and Fermanian, 2001; Matthey et al., 2013). A recent Nepalese study tended to show that the scale was also applicable to Asian infants (Ulak et al., 2020). The results show that about 16.7% of the children were socially withdrawn. Compared with those without social withdrawal, children with social withdrawal were older and had higher proportions of boys (68.4 vs. 42.1%) and social-emotional development delay (63.2 vs. 0%). In age-specific analyses, social-emotional development was poorer in children with social withdrawal across all age groups from 3 to 24 months. Assessed by the ADBB, the prevalence of social withdrawal tendency in young Chinese children was similar to that reported in the European population. Chinese children with social withdrawal tended to have poorer social-emotional development. Two studies in Africa have also demonstrated some transcultural validity (Durand, 2014, in Capetown; Okitundu-Luwa et al., 2021 in Kinshasa), yielding high levels of ISSWB in high-risk samples. In the more recent one, 458 mother-infant dyads were recruited in the city of Kinshasha's (Democratic Republic of the Congo) public mother and child health-care centers. The eight items of the Alarm Distress Baby scale (ADBB) and the five items of modified ADBB (m-ADBB) were used to assess sustained withdrawal behavior (ISSWB). The Goldberg Depression and Anxiety Scales were used to assess maternal affectivity and mental well-being. A specially designed questionnaire was used to identify stressful events faced by the mother during pregnancy. Using the m-ADBB, the study found a striking figure of 69.2% for ISSWB with ADBB (range 0–29) and 72.7% with the m-ADBB (range 0–10). ISSWB was linked to negative maternal affectivity and to high incidence of stressful events for the mothers, and to the child being viewed as “difficult” by the mother. Positive prenatal affectivity was a protective factor of ISSWB (OR 0.46).

### Early Mother-Child Interactions Adversity, Shared Pleasure and Infant Sustained Social Withdrawal Behavior

Infants in lower middle-income countries are often exposed to early adversities, which may lead to suboptimal caregiving environments and place them at risk of not achieving their developmental potential. Synchrony and positive engagement in the mother-infant relationship plays a critical role in buffering the impact of early adversity. Shared pleasure (SP) is considered a marker of high intensity positive interaction and may hold a promise of improving developmental outcomes (Puura et al., 2019). A remarkable prospective observational study of babies born from mothers with and without mental illness in South Africa recently confirmed the high prevalence of ISSWB in the former (Lachman et al., 2021), as well as the buffer effect of shared pleasure against negative influences. Dyadic videos of 91 mother-infant interactions were assessed for shared pleasure (SP) and infant withdrawal (using the Alarm Distress Baby Scale) at 6 months. Infant developmental outcomes were assessed using the Bayley's Scales for Infant and Toddler Development, third edition, at 18 months. The occurrence of SP was low (20%). Importantly, there was no significant relationship between EPDS measure of maternal depression (*p* = 0.571) and Shared Pleasure moments. Being postnatally depressed does not mean being withdrawn most of the time. In fact, most Postnatal Depressed mothers seem to channel all available energy into the relationship with their infant. Infant withdrawal was high (72%) and associated with male infant gender (*p* = 0.025). There was a significant correlation between the occurrence of Shared Pleasure and a lower score of infant withdrawal (estimate = −1.29; SE = 0.4; *p* = 0.0002). The number of Shared Pleasure moments at 6 months was significantly associated with composite motors scores (estimate = 2.4; SE = 0.9; *p* = 0.007) and marginally significant with relation to cognitive scores (estimate = 1.9; SE = 1.0; *p* = 0.052) at 18 months. Regression modeling differential outcomes showed greater improvement in cognitive scores at 18 months in infants with an Shared Pleasure moment compared to those without an SP moment [SP average difference (AD) = 7.4 (2.4), no SP AD = 10.4 (1.2); *p* = 0.012]. Infants without an SP moment experienced a larger decrease in motor scores at 18 months compared to those with an SP moment [SPAD = −3 (3.0); no SP AD = −10.6 (1.5), *p* = 0.027]. While the occurrence of SP in this sample was low and the rates of infant withdrawal were high, there were promising results suggesting that early positive SP interactions may contribute to improvements in subsequent developmental outcomes. The buffer effect of shared pleasure is in line with the study by Sharp et al. (2012), who reported that frequency of infant stroking reported by mothers moderates the effect of prenatal depression on infant behavioral and physiological outcomes. A general population sample of first-time mothers was recruited for assessment at 32 weeks based on reported inter-partner psychological abuse, as a risk to prenatal stress and depression for mothers. Mothers reported how often they stroked their babies at 5 and 9 weeks. At 29 weeks, assessment of respiratory sinus arrhythmia as a measure of the vagal tone (withdrawal to a stressor) was used as a measure of physiological adaptability (Moore and Calkins, 2004). There was a significant relationship between prenatal depression and maternal stroking in the prediction of vagal reactivity to a stressor (*p* = 0.01) as maternal reports of infant anger proneness (*p* = 0.007) or fear (*p* = 0.043). Increasing maternal depression was associated with reduced physiological adaptability and higher negative emotionality, but only in the presence of low maternal stroking.

### Infant Social Withdrawal, Maternal Major Depression, and Trauma

Over the past decades, numerous studies have contributed to a better understanding of maternal depression and its potentially adverse impact on child development (Evans et al., 2012). Research has shown that mothers with depression frequently have difficulties in scaffolding their infants' emotional needs (for a review, see Tronick, 2007). Infants might subsequently develop sustained social withdrawal behaviors to cope with their suboptimal parenting environment (Feldman, 2007). Unfortunately, this coping strategy can set a vicious cycle in motion, as depressed mothers might interpret their infant's withdrawn behavior as “rejecting” and subsequently have even more difficulties in engaging with them (Feldman, 2007).

Despite the clear relationship between maternal depression and infant withdrawal behaviors, studies of the relationship between infant ADBB scores and depression have been mixed. On one hand, several studies have demonstrated significant correlations between positive ADBB scores and indicators of maternal depression, such as maternal depressed parenting behaviors and maternal depressive symptoms in the postpartum period (Dollberg et al., 2006; Matthey et al., 2013). Regarding current depressive symptoms, only one study to date has found significant associations (Mäntymaa et al., 2008). Four other studies examining relations between maternal depressive symptoms at the time of the assessment and infant ADBB scores did not find relationships (Dollberg et al., 2006; Matthey et al., 2013; Guedeney et al., 2017). Importantly, in these studies, presence of depression was assessed based on screening instruments rather than psychiatric diagnostic processes. In the ADBB validation study, 155 mother-infant dyads were evaluated at the 6-month primary care visit. Maternal depression was determined based on a psychiatric interview. Infant social withdrawal behavior was assessed using the ADBB; Guedeney and Fermanian, 2001) based on videotaped mother-infant interactions. Of this sample, 18.7% of mothers were diagnosed with Major Depressive Disorder (MDD), and 39.4% of infants scored above the clinical ADBB cut-off. Infants of depressed mothers were more likely to score positive on the ADBB (75.8 vs. 31.0%, *p* < 0.001) and showed distinct patterns of withdrawal behavior. Within the group of withdrawn infants, however, no differential patterns of behavior could be identified for infants of depressed mothers as compared to infants of mothers with no depression. These findings confirm the validity of the ADBB for detection of infant social withdrawal in the context of Major Depressive Disorder. At the same time, they support evidence that the ADBB identifies non-specific infant distress behaviors.

Recently, Burtchen et al. confirmed the strength of the relationship between Major Depressive Disorder and Infant Sustained Social withdrawal behavior (Burtchen et al.^1^). One hundred ninety-eight women and their 6-month-old infants were studied in a high-risk community sample. ISSWB was assessed using the ADBB, whereas maternal trauma and associated psychopathology were assessed in a psychiatric interview. Atypical maternal behavior was assessed using the Atypical Maternal Behavior Instrument for Assessment and Classification (AMBIANCE) as developed by Lyons-Ruth et al. (1999). AMBIANCE measures five dimensions of maternal behavior: negative-intrusive behavior, role confusion, disorientation, affective communication errors, and avoidance/withdrawal. Maternal trauma was correlated with increased atypical maternal behavior and increased infant social withdrawal (*p* ≤ 0.001). Maternal post-traumatic stress disorder (PTSD) alone, major depressive disorder (MDD) alone, and co-morbid PTSD/MDD were predictive of increased atypical maternal behavior (*p* ≤ 0.001) but only maternal MDD was predictive of infant withdrawal (*p* ≤ 0.001). This study confirms that atypical maternal behavior is an important target behavior for early intervention to improve both maternal and infant well-being. As Tereno et al. (2017) had shown in the large CAPDEP controlled intervention study on a high-risk sample through home visits, enhanced preventive intervention significantly decreased the rate of disorganized behavior as measured using AMBIANCE and decreased social withdrawal behavior as measured using the ADBB (Guedeney et al., 2013), despite the fact that postnatal depression in this sample (30%) was not significantly reduced. Both Burtchen‘s study and the Tereno CAPDEP study are in line with Okintungu et al.'s Kinshasha ADBB study quoted above: high levels of maternal stress and maternal depression yield high levels of ISSWB, leading to an alteration of the child's developmental outcomes. These initial findings in humans indicate that maternal stroking in infancy, as reported by mothers, has effects strongly resembling the effects of observed maternal behaviors in animals, pointing to the need for future studies of the epigenetic, physiological, and behavioral programming of hypothalamic-pituitary-adrenal function and health (Meaney et al., 2007).

### Infant Sustained Social Withdrawal Behavior and Organic Illness: Congenital Heart Disease, Prader-Willi Syndrome, Cleft Lip and/or Palate Syndrome

#### ISSWB in Infants With Congenital Heart Disease (CHD)

In the context of major organic illness in infants, including congenital heart disease (CHD) where surgery in infancy is often required, previous studies have noted high levels of stress in parents. Symptoms of depression and anxiety or trauma reactions have been observed in mothers of children subjected to cardiac surgery. Sam Menahem, from the Pediatric Cardiology Unit, Monash Medical Centre, Melbourne, Australia, and Jennifer Re were among the first to study infants' psychological responses to early cardiac surgery for life threatening cardiac conditions. The aim of their study was to review infant responsiveness using the ADBB scale as a standardized objective observational measure of social withdrawal and to explore its association with measures of maternal distress (Edinburgh Postnatal Depression Scale, Spielberg State-Trait Anxiety Scale and Parenting Stress Index-Short Form). This study involved 22 Australian mothers whose infants-−2 months old or older–underwent cardiac surgery for congenital heart disease (CHD), which included the full spectrum of the more severe Congenital Heart Disease abnormalities. High levels of infant sustained social withdrawal behavior and maternal distress were observed when the mothers and infants were assessed. Using the ADBB scale, 10 of the 22 infants were socially withdrawn, 6 of them recording very high scores suggesting severe withdrawal. The elevated ADBB scores also suggested that these infants'psychic well-being was at risk beyond their physical well-being. The ADBB scores were among the highest levels reported in the literature. Interestingly, infant sustained social withdrawal behavior was not significantly associated with the severity of the Congenital Heart Disease and the complexity of the surgery required. The effect of maternal distress in such a situation on the mother-child interaction is likely to be intermediated by oxytocin (OXT). One would expect mothers with the highest levels of OXT to be the ones whose infants have the lower level of ISSWB. Indeed, a recent study by Lisanty (2021) demonstrated the effects of skin-to-skin contact (SSC) on biobehavioural measures of stress (anxiety and salivary cortisol) and attachment (attachment scores and salivary oxytocin) in mothers before and after their infants' neonatal cardiac surgery. Theses biobehavioural markers studied increasingly more in perturbed parent-child relationship situations, whether induced by somatic adversities or not.

### The Prader-Willi Syndrome: ISSWB, Dys-synchrony of Parent-Infant Interaction and Anorexia Reduced by Postnatal Intranasal OXT Administration in 6 Months Old PWS Infants

Oxytocin (OXT) is a neuropeptide that plays an important role in modulating social interactions and mother-infant bonding (Numan and Young, 2016). Post-mortem human hypothalamic tissues from patients with Prader-Willi syndrome (PWS) have demonstrated a reduced number and volume of OXT neurons in the paraventricular nucleus in comparison with controls (Swaab et al., 1995). This observation confirmed the origin of the symptoms in adult Prader-Willi patients suffering from severe bulimia and of regulation disorders due to the lack of OXT in the midbrain. PWS is a rare genetic disease caused by the lack of expression of paternally inherited imprinted genes on chromosome 15q11-q13 due to a deletion of chromosome 15q11-q13, maternal uniparental disomy, or an imprinting defect. This complex neurodevelopmental disease comprises several nutritional phases. From birth to 9 months, infants with Prader-Willi Syndrome display severe hypotonia, poor interactions, and anorexic behavior with poor suck (Miller et al., 2011). Genetic diagnosis, which is now made in the 1st months of life, offers a unique opportunity for early treatment with OXT. Tauber et al. conducted a phase 2 study of a short course of intranasal OXT (7 days) administered to 18 infants with PWS under 6 months of age effects on safety, feeding, and social skills, ghrelin levels, and brain connectivity. Sucking and swallowing were evaluated before the first and after the last OXT administration. Social withdrawal behavior and mother-infant interactions using the ADBB scale and the validated Coding Interactive Behavior (CIB) scale were independently assessed using the feeding video tapes (Viaux-Savelon et al., 2014). At baseline, the median ADBB score was 6.5, with 62% of the infants with an ADBB score of ≥ 5. The median score significantly improved for the whole group from 6.5 to 3.5 (*P* = 0.005), with a normal score in 81% of infants after OXT treatment. To our best knowledge, this study is the first to describe the social withdrawal behavior of 6-month-old PWS infants and its decrease with intranasal OXT, along with the increase of Coding Interactive Behavior sub scores in parental sensitivity from a median score, dyadic reciprocity, child social engagement. A significant ISSWB (ADBB score equal to or over 5) is therefore a salient feature of the early PWS picture. A multisite phase 3 study is ongoing, initiated by the same team, with treatment starting at 1 month of age.

### The Impact of Having a Baby With Cleft Lip and Palate on Parents and on the Parent-Baby Relationship

Grollemund et al. (2012) realized the first study using the ADBB to describe the impact of cleft lip and palate early or later surgery and timing of diagnosis (ante- or postnatal) on ISSWB in a large prospective multicentre study involving 158 infants who had a cleft lip with a cleft palate (CLP) or without one. Their main objective was to explore the effect of the malformation (CLP) on the infant's social withdrawal, the parent's mental health and the parent-infant relationship. Social withdrawal behavior was evaluated by the ADBB scale during follow-up consultations. The parents' mental health was assessed by the Parenting Stress Index (PSI), the Edinburgh Postpartum Depression Scale (EPDS) and the Impact on Family Scale (IOFS). All evaluations were made at 4 and 12 months after the infant's birth. The incidence of social withdrawal among infants with CLP was 13% at 12 months of age, which is the same level as that observed in community studies in France. The authors noted that the ADBB scores decreased from 4 months to 12 months. Interestingly, Grollemund et al. (2020) found that the timing of surgery or the type of malformation did not influence the level of infant social withdrawal behaviors at 4 and 12 months postpartum. They also observed higher postpartum depression scores in both mothers and fathers of infants with CLP compared to the general population at the 4- and 12-month assessments. Interestingly, parents who had been informed of a prenatal diagnosis of CLP were better prepared to accept the waiting time between birth and the first surgical procedure in comparison with parents who learned about the CLP diagnosis at birth. Furthermore, when surgery was performed early (during the first 3 months of age), maternal distress decreased significantly at the 12-month assessment point compared to cases where surgery was performed later.

### Fetal Alcohol Syndrome/Partial Fetal Alcoholic Syndrome and ISSWB

A groundbreaking study by Molteno et al. (2014) in Capetown, South Africa, showed the high level of ISSWB in 6-month-old infants exposed prenatally to alcohol consumption. The sample consisted of Cape Colored (mixed ancestry) infants whose mothers were interviewed during pregnancy regarding their alcohol consumption using a timeline follow back approach. Infant sustained social withdrawal behavior (*n* = 85) was assessed on the ADBB scale at 6.5 months. Mother-infant interaction was evaluated from video recordings during free play and infant feeding at 6.5 months (*n* = 127). Socio-demographic and psychological correlates of maternal alcohol use, infant temperament, maternal postnatal depression and infant iron deficiency were examined as potential confounders. Prenatal alcohol exposure was related with increased infant emotional withdrawal and decreased activity, but unrelated to mother-infant interaction, to mothers' post-natal depression or to any other temperament measures. Children later diagnosed with Fetal Alcohol Syndrome **(**FAS) and Partial Fetal Alcoholic Syndrome (PFAS) at 5 years exhibited a higher level of ISSWB as infants without FAS or PFAS. When all infant affective measures were examined simultaneously using regression analysis, only Infant Sustained Social Withdrawal Behavior persisted as a significant predictor of 9-year intelligence quotient (IQ). This study is therefore the first to document a direct effect of fetal alcohol exposure on ISSWB in infancy. These data link prenatal alcohol to a specific aspect of infant affective function not attributable to mother-infant interaction, infant temperament, or other socio-emotional aspects of the infant's environment and identify infant emotional withdrawal as an early indicator of affective disturbance, particularly in children later diagnosed with FAS and PFAS.

### Assessing ISSWB of Out of Home Placed Infants During Parental Visitation

The impact of children's interactions with parents in the context of out-of-home placements is receiving much-needed cross-disciplinary attention. Child welfare services in Europe and North America continue to grapple with the controversies related to establishing and maintaining child-parent relationships when parental care has been temporarily or permanently suspended. Nowhere is this difficulty more pronounced than in situations of parental visitation, prompting professionals to assess and balance children's need for protection with that of preserving a relationship with primary caretakers (Miron et al., 2013). However, the paucity of instruments that can reliably represent young children's experiences of such interactions precludes a nuanced evaluation of their impact on wellbeing and development. In response to this empirical gap, the study by Deprez et al. (2018) investigated children's relational withdrawal as a clinically salient, easily observable, and conceptually valid measure of infants' and toddlers' responses to parents. Relational withdrawal, challenging behaviors and salivary cortisol were assessed before, during and after parental visits. Findings suggest that observations of relational withdrawal correlate meaningfully with measures of neurobiological reactivity. Clinically, three profiles of cross-variable responses in children appeared, distinguishing between groups that experience increased, decreased, or unchanged levels of stress in response to parental visits. Taken together, the findings lend empirical support to systematic observations of relational withdrawal to bolster evaluations of young children's experience of parental visitation during out-of-home placements.

Thus, ISSWB seems to be as universal a behavior as attachment is. Every newly born infant has to face the challenges of regulating his internal states and stress, using a more or less mature Autonomic Nervous System (ANS) to do so, with the help of a more or less attuned and synchronizing caregiver acting as a buffer between the infant and stress factors. ISSWB therefore seems to be the behavioral correlate of an impaired social engagement system. An increasing literature using the polyvagal theory, highlights the link between ISSWB behavior and the adaptation of the autonomic nervous system to promote survival in an adverse context.

## Neurobiology, Animal Models and Genetics of Social Engagement and Withdrawal Behavior

### Animal Models of ISSWB

The link between depression, withdrawal reactions in infants and learned helplessness behaviors was suggested relatively recently. In a famous, though ethically debatable, experiment by Seligman et al. (1979), a dog was given an electric shock in a situation where there was no escape. Seligman referred to this situation as “learned helplessness,” which led the dog to resignation. The model of learned helplessness has since become a model for depression, and the learned helplessness paradigm eventually became a key screening test for antidepressant activity (Seligman et al., 1979). Panksepp recently suggested a pattern for the main types of emotional systems in mammals: lust, care, panic, play, fear, rage, and seeking. Withdrawal behavior was conceptualized as part of the panic and fear systems (Panksepp, 2006). Therefore, the approach/withdrawal behavioral system is fundamental in the analysis of behavioral development (Greenberg, 1995).

### Attachment, Emotional Regulation and ISSWB

In Bowlby's observations of young children separated from their caregivers in 1973, the gradual decline in attachment behaviors was attributed to a defensive strategy that protects the child from experiencing the unbearable mental pain caused by the absence of maternal care and the breakup of the affective bond (Ainsworth and Bowlby, 1991). On a micro-analytical level, the “still face” experiment (Cohn and Tronick, 1983) shows the same mechanism when a baby's expectations in a relationship are not met. From these observations we can understand that an infant's adaptation to a suboptimal or adverse experience is as follows: when faced with separation, loss or stress (no reaction from the caregiver, generating stress) a human baby will first signal, protest, cry and expect an answer. If no reparation comes from the environment and especially from the attachment figure, then the human infant will display a new set of behaviors. As early as 1973, Bowlby stressed the importance of the balance between attachment behaviors and withdrawal behaviors, with two pages on the topic in *Separation: Anger and Anxiety*, in the chapter on forms of behavior indicative of fear (Bowlby, 1973, p. 115–117). Bowlby classified attachment behavior in the broader category of "fear behaviors (…). It is obvious that, if confusion is to be avoided, distinctive names are also required for any other components of fear behavior that can be clearly identified. For behavior that tends to increase the distancing from people and objects that are treated as though they were threatening, the terms “withdrawal,” “escape,” and “avoidance” are all suitable. For another principal and well-organized component, namely behavior that results in immobility, the usual term is “freezing” (p. 115). Bowlby made the following suggestion: “attachment behavior and withdrawal behavior are distinct behavioral systems that (a) have the same function, (b) may be elicited by many of the same conditions, (c) are frequently compatible with each other, but (d) can easily be in conflict. In cases of conflict, it is a matter for inquiry to discover which, if either, takes precedence” (Bowlby, 1973, p. 117).

### Attachment as the Major Physiological Buffer Against Stress

Many studies have explored the physiology of infant attachment, according to Myron Hofer's description of the attachment system as a “hidden regulator” in rodents (Hofer, 1995; Opendak et al., 2020). Most of them involved assessments of infants' physiological reactions during Ainsworth's “strange situation.” The levels of salivary cortisol in infants with disorganized attachment remain very high even after reunification with the attachment figure. Infants with a resistant (“angry”) style of attachment exhibit a curve close to the disorganized curve, albeit with lower levels of cortisol. Avoidant attached infants show a low level of cortisol in the first phases of the procedure, but the delayed elevation of cortisol remains high longer after elevation, then slowly decreases, indicating that the infants are still stressed by the separation despite not showing any external sign of distress. Secure infants display a clear initial increase in salivary cortisol and in mean heart rate, but a rapid decrease shortly after they are reunited with their caregivers (Spangler and Grossmann, 1993). These studies confirm the psychobiological basis of attachment theory, which stresses the roles of regulatory input and the dyadic bio-behavioral attunement of the attachment figure in the development of the child's self-regulatory abilities.

Concurrent correlations between mother-infant synchrony and cardiac vagal tone were observed by Moore and Calkins (2004). Infants who engaged in synchronous interactions with their mothers showed greater vagal brake during the still-face phase that followed. Vagal brake measures change in vagal tone, from a calm to a stressed state, and a more marked brake indicates a more adaptive systemic adjustment to environmental intrusions. Gunnar suggested that a function of secure attachment could be protection against the negative effects of a sustained, elevated level of glucocorticoids on the developing brain as a consequence of exposure to stress (Gunnar et al., 1996). In 2005, Zelenko neatly showed that secure dyads demonstrated more consistency of dyadic heart rate changes than insecure-resistant dyads (Zelenko et al., 2005). Theses mechanisms need to be consolidated by further studies.

### ISSWB's Genetic Susceptibility: The Research for Candidate Genes

As Insel puts it in his 2010 review, social neuroscience is still a frontier area of neurobiology. One of the most exciting areas of this frontier is the opportunity to bridge the insights emerging from studies of social cognition and social behavior in animals to human research. While there is a temptation to translate “animal models” of human disorders or to assume that findings in animals will map directly on to human neurobiology, the translational bridge will need to be built with careful consideration of species differences, based on evolutionary adaptations (Bartz and Hollander, 2006). While some of the principles may be conserved (i.e., the importance of receptor maps and the role of gonadal steroids), the details for social organization need to be explored for each species, recognizing the importance of diversity in the neural mechanisms for social cognition (Insel, 2010, p. 11). Hence the importance of joining facts, concepts, and hypotheses stemming from developmental research with the ones stemming from the best clinical controlled studies, if one attempts to overcome the challenge of translation in social neurosciences.

ISSWB is a feature of most major psychopathological diagnostic categories in 0–3 year-old infants, and above all in infants with autism spectrum disorder (ASD) (Ozonoff et al., 2010). The first candidate gene for ISSWB are the ones found to be linked with ASD. ASD and PWS have a common clinical feature—ISSBW—and therefore probable common susceptibility genes. Mouse models have demonstrated that alterations in the OXT system play a major role in the PWS. Interestingly, a single OXT injection before the first 5 h of life rescued 100% of the newborn Magel2 knock-out (KO) mice from early death by restoring normal sucking activity. The Magel2 KO mouse is now considered a mouse model for PWS and ASD because truncated mutations in the Magel2 gene have been reported in some patients with ASD (Schaller et al., 2010; Schaaf et al., 2013). Altogether, these data suggest that OXT is involved in the pathophysiology of PWS and ASD. A major goal of developmental research is now the search for early valid signs of increased risk of ASD. A number of researchers have suggested that oxytocin and vasopressin may be implicated in the etiology of autism given that deficits in social interaction and affiliation are a core feature of autism and that these neuropeptides are involved in the regulation of affiliative behaviors (Insel, 2010). Controlled and longitudinal studies of infants born into families with pre-existing ASD has tremendously helped the recognition of the early signs of autism and their order of appearance (Zwaigenbaum, 2021). Based on their own groundbreaking longitudinal study (Ozonoff et al., 2010), Ozonoff recommended paying further attention to longitudinal assessments in clinical screening, that is, the use of trajectories rather than cross-sectional differences as early detection metrics. This approach can lead to earlier detection and, accordingly, the opportunity to intervene at an earlier stage of functional decline.

From this perspective, ISSWB appears to be an interesting behavior to use in early screening for ASD in infants, particularly to explore the period between birth and 12 months of age (Guedeney, 2019). Two concepts may be useful in this respect, as both have strong links with parent-infant synchrony in the first 18 months of life (Feldman, 2007). The first and more specific one is the affect attunement concept, based on the intermodal matching abilities of the neonate, and the second, less specific one is the infant's sustained social withdrawal concept. Falck-Ytter et al. (2018) designed an elegant study with 10-month-old siblings of children with autism using an eye-tracking task. These authors found that infants who later received an autism diagnosis did not orient to audiovisual synchrony expressed within biological motion, suggesting that reduced orienting to audio visual synchrony within biological motion may be an early sign of autism. They judiciously added that poor multisensory processing could be an important antecedent marker of autism. Falck-Ytter et al. cautiously refrained from pushing forward their findings into a hypothesis about the possible causal origins of this alteration of audiovisual synchrony, as observed at 10 months of age (Meltzoff and Borton, 1979). It seems that some connection could be made here with the concept of intermodal matching described in 1979 by Melzoff and Borton. In a now very famous experiment, 1 month-old infants showed clear preferential looking toward the replica of the object they had just explored orally. Meltzof and Borton concluded that intermodal matching is well-established at this age, favoring the notion that there is an innate unity of the senses. Following on Meltzoff and Borton's groundbreaking study, Daniel Stern, a developmental researcher, demonstrated how transmodal ability seems essential to the normal development of secondary intersubjectivity, as it enables the process of affect attunement between mother or father and child to unfold (Stern, 1985). Attunement involves intermodal resonance between mother (parents) and infant, through (a) parental matching (not necessarily imitating) of the infant's internal feeling state, (b) cross-modal affective expression between parent and infant, and (c) demonstration of behavior that expresses the quality of a shared affect state. The point here is that intermodal matching allows for affect attunement, which in turn allows an increase in the infant's ability to get deeper into an intersubjective relationship, in a truly dyadic process. Both intermodal matching abilities, which are observed from birth onward, and affect attunement, a capacity observed around the age of 7/8 months, develop within the parent-infant relationship, and particularly within the interactional synchrony between parent and infant in the first 18 months of life (Feldman, 2007). The lack of synchronization of gaze, as early as 6 months of age, seems to be among the earlier stages of the development of ASD as shown by Ozonoff et al. (2010). Around the age of 7–8 months, the difficulty experienced by the parent to synchronize (gaze, vocals movements) with the child to be subsequently diagnosed with ASD is in fact a lack of affect attunement (Guedeney, 1997a,b, 2000). Indeed, a comparative and controlled study of family films using the ADBB shows that the ADBB score at 6 months of age is predictive of an ASD diagnosis at 3 years of age. These examples show how translation may help cross-fertilization in developmental research.

## Survival Comes at a Price: The Link Between ISSWB and Adversity in Light of the POLYVAGAL Theory

Researchers have attempted to identify Biomarkers associated with emotion regulation. Del Giudice et al. (2011) suggested an evolutionary-developmental theory of individual differences in the functioning of the stress-response system. In this theory, the stress-response system has three main biological functions: (1) to coordinate the organism's allostatic response to physical and psychosocial challenges; (2) to encode and filter information about the organism's social and physical environment, mediating the organism's openness to environmental inputs; and (3) to regulate the organism's physiology and behavior in a broad range of relevant areas, including defensive behavior, competitive risk-taking, learning, attachment, affiliation and reproductive functioning. The information encoded by the system during development provides feedback on the long-term calibration of the system itself, resulting in adaptive patterns of responsiveness and individual differences in behavior via the development of a “life history strategy.” Del Guidice's Adaptive Calibration Model (ACM) is a powerful tool for understanding the evolutionary meaning of individual differences and for analyzing them within the framework of strategic variations (Del Giudice et al., 2011). Different phenotypes can be conceptualized as the manifestation of different adaptive strategies, that is, ways for an organism to weigh up the costs and benefits in order to maximize its expected trans-generational fitness. For Del Giudice, it is conditional adaptation that redirects the life strategy at different developmental points and leads to the expression of one phenotype or another. During the 1st year of life, functioning as an organism under stress, the child gathers information about his environmental ecology (morbidity-mortality, unpredictability, available resources) both directly and through parental behavior. We thus support the hypothesis that ISSWB could appear when the infant organism collects information signaling a complex and potentially dangerous environment, triggered by the absence of regulation of internal states and of the nervous system that normally occur within the attachment relationship with the parent caregiver. The activation of an psychic economic strategy of expectation, i.e., relational withdrawal, liable to be revised at the next developmental point, could be a conditional adaptation, a compromise that will maximize survival in an adverse context by reducing exploration and the associated dangers, as well as crying and demands, in order to avoid triggering aggressive responses, while limiting as far as possible the energy expenditure in an environment recognized by the organism as being unpredictable and potentially dangerous. ISSWB could therefore be a developmental phenotype activated as a life history strategy in the 1st years of life when infants are confronted with adversity.

In an other hand, considering the long term developmental consequences of exposition to fear and trauma, studies by Opendak et al. (2019, 2020) demonstrate the key role of maternal regulation: “maternal presence blocks fear learning and amygdala plasticity through age-dependent suppression of amygdala *AMPA* receptors (receptors activated by glutamate) subunit trafficking, maternal presence suppresses engagement of brain regions within the mesolimbic dopamine circuit, and early-life abuse compromises network and molecular biomarkers of maternal regulation, suggesting reduced social scaffolding of the brain” (Opendak et al., 2019, p. 1247). Using a naturalistic rodent model of maternal maltreatment during a sensitive period, postnatal days 8–12, to examine social behavior in infancy, peri-weaning and adolescence, Rincón-Cortés and Sullivan (2016) provide data on the cortico-limbic involvement in the emergence of maltreatment-induced social deficits that are linked to adult depressive-like behavior, thereby highlighting potential targets for therapeutic intervention.

The maturation of the parasympathic branch of the Autonomic Nervous System (ANS) has been identified as a critical factor in supporting the development of increasingly sophisticated behavioral regulation processes (Moore and Calkins, 2004; Porges, 2007).

Polyvagal theory (Porges, 2007), is regarded as the most influencal model in differentiating the relation between vagal tone during steady states (i.e., baseline vagal tone) and vagal reactivity (ie vagal regulation) in response to environmental challenge. Its mechanisms are complex and need to be further explored as done in recent laboratories studies (Levine et al., 2016; Mueller et al., 2021). The polyvagal theory states that the autonomic nervous system (ANS) reacts to challenges experienced by the child in a predictable, hierarchical manner that mirrors the phylogenetic history of the ANS in vertebrates and provides insight into how the human body adapts and responds to stress and danger (Porges, 2011). The ANS is a division of the peripheral nervous system which supplies smooth muscles and glands, and thus influences the function of internal organs. This control system acts largely unconsciously, regulating bodily functions such as heart rate, digestion, respiratory rate, pupillary response, urination, and sexual arousal. Polyvagal theory emerged from research on heart rate patterns in the human fetus and newborn babies. It was intended to resolve the paradox of cardiac vagal tone being at the same time a sign of risk status and of resilience. Porges's conception of the ANS, as being organized in three phylogenetic pathways with existence of a myelinated mammalian vagus, were intended to solve the paradox.

For neonates, maintaining physiological homeostasis is crucial for survival. The vagal system contributes to the regulation and coordination of the survival process, including breathing, sucking, swallowing, heart rate and vocalization (Porges, 2011, p. 89). Studies by Porges and colleagues (Doussard-Roosevelt et al., 1997) indicated that the vagal tone in babies was low under the age of 3 months, and in some infants up to 6 months. This has a profound effect on the way the nervous system responds to situations and stimuli. When the vagal tone is low, the ability of the nervous system to “correct” itself when out of balance is hampered. As infants have no way of inhibiting the fight-or-flight response on their own, they may become disorganized and be endangered if no adult takes action for them. Research on other species has demonstrated that cardiac vagal tone increases during development, along with self-regulatory and exploratory behaviors (Porges, 2011, p. 73). This means that the development and maturation of these adaptive systems both endow infants with new skills and help them face challenges and stress as time passes. The mammalian vagus is only partially myelinated at birth and continues to develop during the 1st month postpartum (Porges, 2011, p. 122). Given human babies' immaturity at birth, the need in the 1st year of development is to regulate body function and affective states and to promote social engagement. It is also to achieve some level of synchronization between infant and caregiver, moving from an emerging ability for self-regulation to a more dyadic regulation process (Feldman, 2007). Only an attachment figure can help the infant learn to regulate over time. It is the attachment relationship that ultimately organizes the developing Autonomic Nervous System and especially the mammalian vagus, enabling better adaptation to stress, emotional regulation, and mental health later on in life. Therefore, co-regulation from a caregiver is of paramount importance for survival, and if it proves inadequate, it can trigger survival responses based on a more primitive neurophysiological system: the fight-or-flight response (distress and protest) and the freeze response, merely a conservational system to conserve energy and save time (Engel and Schmale, 1972), since human infants require a conservational, energy-saving system to face relational adversity in the 1st months of life when the attachment and social engagement system does not yet provide sufficient regulation. Porges (1995) hypothesized that the emergence of behaviors and mental states was related to affiliation and parenting, and that intimacy was linked to the development of the polyvagal system through the evolutionary process. This development marks a shift from sympathetic-adrenal control of the heart rate to the graded system observed in mammals, which enables quick changes in metabolic inputs and outputs from the heart and facilitates complex behavioral types, such as attention, orientation, and calmness required for bonding. The removal of the vagal brake through signals from the vagus via myelinated pathways that originate in the nucleus ambiguous enables a rapid increase in heart rate in response to external or internal stressors and better adaptation to changing environmental inputs, and hence a more competent adaptation to micro-shifts in affective facial and postural cues, which are abilities that underpin synchrony (Feldman, 2007). Recent studies in functional imagery have reinforced the idea of a differential cost of these different regulation strategies using different networks. The lack of sufficient experience of being regulated by someone else leads to an increase in amygdala activation, through a more costly regulatory pathway, through the prefrontal dorso-lateral cortex. When faced with images of distress, individuals with resistant styles of attachment tend to show more amygdalian activation than more secure individuals, while avoidant individuals show more prefrontal and dorso-lateral cortical activation (Vrticka et al., 2012).

### ISSWB as the Behavioral Correlate of Impaired Vagal Brake

We suggest that ISSWB is an observable, defensive behavior in the fear and freeze repertoire opposed to “fight-or-flight” behaviors. This latter one is based on the generalization of a freezing reaction, which could stem from the activation of the dorsal (most ancient) part of the vagus nerve by acute or chronic stress without sufficient regulation from a safe and reliable caregiver. Human infants, more than any other mammalian babies, could in this view rely much more on the social engagement system based on the mammalian vagus system if sufficient support is available, and much more on the immobilization system based on the dorsal reptilian vagus system if resources and relationship are lacking. Indeed, the mobilization system requires months to be fully available as a useful strategy for surviving danger.

If we consider that ISSWB develops from an infant's disengagement skills acting in response to a pervasive lack of attunement and de-synchronization (lack or absence of repair), we then have to recognize it as a sign of failure of the attachment system. Pervasive activation of the immobilization system could be the underlying physiological base for the onset of ISSWB as a freezing reaction, which may be the only solution left to an infant. When faced with a threat of this sort, a vulnerable being such as an infant has to call on the immobilization system. The unmyelinated vagus and the freeze/death-like, energy-conservation strategies seem effective in promoting survival: playing dead to save energy, waiting for the environment to change for the better, waiting for other developmental resources to appear, without signaling one's presence to potential predators.

### The Social Engagement System and Social Withdrawal Behavior: Two Ends of the Same Dimension?

ISSWB seems to play a similar role to what is seen when the social engagement system shuts down. Indeed, Porges (2011, p. 46) reported that the social engagement system includes the regulation of the eyelids through the orbicularis oculi (control of social gaze and gesture), the muscles of facial expression (control of emotional expression, smile, frown), middle ear muscles (distinguishing human voices from background sound), mastication muscles (feeding, sucking), laryngeal and pharyngeal muscles (vocalizing, swallowing, breathing) and head-turning and tilting muscles (social orientation). This link between face, eye movement, detection of sound, especially the human voice, and vocalization on the one hand and vagal states (and therefore neurophysiological states) on the other hand, i.e., the absence or presence of stress, gives us information about the internal experience of the baby. If infants can direct their gaze, vocalize, smile, and engage, their physiological state is good and the social engagement system is active, which means that on a regular basis, the attachment system is working sufficiently well, and the environment is sufficiently welcoming. As they encounter more stressful states, infants enter the mobilization phase and protest vigorously to try to repair the bond with the caregiver. In doing so, they have more difficulty in directing their gaze at humans, they do not smile, and vocalization is pressing, aimed at attracting care. But protest is a biologically costly defensive behavior that cannot be maintained for a long time.

The immobilization system, or freeze reaction, is the solution when no regulation is forthcoming from the environment. While in a freezing state (even for a few seconds), infants do not look at human faces, or only look at them vacantly, and their face is still, featureless and sad. Babies tend to stop moving or slow their movements. Vocalizing, whether positive or pressing, decreases greatly and is not spontaneous. Infants become mostly silent and appear falsely calm and content. This behavior, significant of a dorsal vagal freeze state, is exactly what is observed in an infant showing strong relational activity.

### Many Different Sensitive Periods for Development Are Still to Be Mapped and Described With Accuracy

As infants transition from dependence on the mother to self-regulation, functional connectivity patterns expand to support new behavioral patterns and newly functionally maturing brain areas (Gilmore et al., 2018; Opendak et al., 2019). However, we know little about these transitions, despite recent evidence suggesting that they are periods of vulnerability to the initiation of pathways to pathology and developmental disorders. Recent OXT research has demonstrated the existence of such a sensitive period for the presence of OXT in the midbrain, during pregnancy and during the 1st year of life, as shown by the effects of OXT in PWS infants (Tauber et al., 2017) and ASD adults.

### Perspectives for Research

It could be very valuable to study the neurophysiological and neuro-imagery correlates of relational withdrawal in nonclinical populations confronted with stress activation, to compare this with infants developing in adverse contexts and check if the permanent withdrawal could be incompatible with the neural network default mode. Following the same line of thought, observing and assessing relational withdrawal via test-retest in relation to attachment classifications and neurophysiological indicators of stress (heart rate, vagal tone, cortisol level), especially in an unfamiliar setting, could yield interesting information on the specific adaptation on which each attachment strategy is organized. Another line of research could be longitudinal, with the aim of demonstrating a link between quality of interaction, attachment organization, vagal tone and maturation of the ANS on the one hand and the evolution of relational withdrawal behavior over the two 1st years of life and later psychopathology on the other.

Considering ASD, it would be interesting to look for commonalities with the genes concerned with the intermodal matching process, when these genes will be identified.

Conversely, it would be relatively straightforward to check whether the level of dys-synchronization between infant and parent is or is not an early sign of ASD, a the decrease in frequencies of gaze to faces, social smiles, and directed vocalizations. This is what we intend to do, applying a new scale for synchronization (Guedeney et al., EASY©; Viaux et al. BIS©) to different groups of clips of parent-infant face-to-face interaction already scored with the ADBB and with the CIB (Coding Interactive Behavior, Feldman and Eidelman, 2003).

Finally, research linking child temperament, genetics, relational withdrawal and vagus functioning is of great importance, because it could provide highly important information about individual differences observed in reaction to stress and adversity and the impact of the environment and genetics on development disorders and resilience, as well as identify the genetic basis of individual differences in genetic susceptibility to SSWR. Recent research with a Canadian sample of 650 mother-infant dyads showed relationships between the DRD4 alleles, lower birth weight and maternal sensitivity, leading to more or less disorganized attachment behaviors in the infants (Wazana and Moss E, 2015), which recalls the relationship between ISSWB and birth weight and gestational age in the EDEN study (Guedeney et al., 2017).

## Conclusion

This paper emphasizes the idea that SSRW could be a sign of risk for infant development in a safe environment while at the same time being an effective, adaptive, short-term strategy that promotes security and survival in an adverse context. SSRW could be maintained in our behavioral repertoire as a phylogenetic resource to promote species survival. It seems to function as an energy conservation system while simultaneously reducing the risk of harm, accident, aggression, and predation in the absence of a trusted or familiar caregiver, by reducing protestation and attachment signals, movement, and exploration. A behavior system of this type seems to have developed on the basis of the infant's innate disengagement skills, with minimized activation of the attachment system and the use of flight/freeze primitive strategies to save energy and wait for better times. ISSWB could thus develop over time in response to the absence of stress regulation, from a very mild disengagement process to massive freeze states. The neurophysiological basis of this behavior could be the ANS and its function and development as proposed by the polyvagal theory. Hence the importance of putting together facts, concepts, and hypotheses stemming from developmental research with the ones stemming from the best clinical controlled studies, overcoming the challenge of translation in social neurosciences (Insel, 2010). Doing so may stimulate valuable cross-fertilization effects between these different disciplines and help us take new steps toward understanding the still very mysterious intricacies of psychopathology.

## Data Availability Statement

The original contributions presented in the study are included in the article/supplementary material, further inquiries can be directed to the corresponding author/s.

## Author Contributions

AD, AG, and SV-S: substantial contributions to the conception or design of the work, or the acquisition, analysis or interpretation of data for the work, and agree to be accountable for all aspects of the work in ensuring that questions related to the accuracy or integrity of any part of the work are appropriately investigated and resolved. AG: drafting the work or revising it critically for important intellectual content and provide approval for publication of the content. All authors contributed to the article and approved the submitted version.

## Conflict of Interest

AD was employed by B-Families Sarl. The remaining authors declare that the research was conducted in the absence of any commercial or financial relationships that could be construed as a potential conflict of interest.

## Publisher's Note

All claims expressed in this article are solely those of the authors and do not necessarily represent those of their affiliated organizations, or those of the publisher, the editors and the reviewers. Any product that may be evaluated in this article, or claim that may be made by its manufacturer, is not guaranteed or endorsed by the publisher.

## Footnotes

^1^Burtchen, N., Alvarez-Segura, M., Urben, S., Giovanelli, C., Mendelsohn, A. L., Guedeney, A., et al. (2022). Effects of maternal trauma and associated psychopathology on atypical maternal behavior and infant social withdrawal six months postpartum. (submitted).

## Abbreviations

ISSWB: Infant sustained social withdrawal behavior
ADBB: Alarm Distress Baby Scale
m-ADBB: modified Alarm Distress Baby Scale
ANS: Autonomic nervous system
ASD: Autism spectrum disorder
CHD: Congenital heart disease
FTT: Failure to thrive
FAS: Fetal alcoholic syndrome
OXT: Oxytocin
KO: Knock-Out mice/gene
MPTSD: Maternal post-traumatic stress disorder
MDD: Major depressive disorder
PWS: Prader-Willi syndrome
PFAS: Partial fetal alcoholic syndrome
RSA: Respiratory sinus arrhythmia
SP: Shared pleasure
SSC: Skin-to-skin contact.
